# A pan-neotropical analysis of hunting preferences

**DOI:** 10.1007/s10531-017-1334-8

**Published:** 2017-04-25

**Authors:** C. A. Stafford, R. F. Preziosi, W. I. Sellers

**Affiliations:** 1 School of Earth and Environmental Sciences, University of Manchester, Manchester, United Kingdom; 2 School of Science and the Environment, Manchester Metropolitan University, Manchester, United Kingdom

**Keywords:** Hunting, Bushmeat, Neotropical forests, Mammals, Species preferences

## Abstract

Hunting in the neotropics is a widespread form of resource extraction. However, there is increasing concern that current activities are leading to the decline and extirpation of vulnerable species; particulary ateline primates, large ungulates (such as tapirs and white-lipped peccaries) and large birds such as curassows. Hunting patterns are expected to be a product of two principal influences: the value of return for a given amount of effort invested into hunting, and cultural factors that determine the prestige and usefulness of prey. Previous work has suggested that hunting profiles change in a predictable way over time, becoming more diverse and more dependent on smaller bodied species as preferred, large-bodied prey become scarcer. In this paper, we evaluate the hunting profiles of 78 neotropical communities in Central and South America. We investigate the uniformity of species preferences, whether communities that are geographically closer have similar hunting profiles, and whether the age and size of settlements can be used to predict the type and diversity of species targeted. We found that there was only a weak correlation between the structure of communities’ hunting profiles and their geographical proximity. Neither a community’s size nor age was a good predictor of the shape and structure of its hunting profile. Our data suggest that either the availability of prey or the cultural influences dictating the value of different species can change rapidly over small distances, and that older and larger settlements do not impact prey species distributions in a predictable way.

**Electronic supplementary material:**

The online version of this article (doi:10.1007/s10531-017-1334-8) contains supplementary material, which is available to authorized users.

## Introduction

Bushmeat hunting in the forests of Central America, Amazonia and the Guianan shield is a widespread form of resource extraction that has historically provided forest communities with an essential source of protein (Marmolejo 2000; Robinson and Redford 1991). How sustainable this activity is has been subject to much debate (Fa et al. 2002; Levi et al. 2011; Shepard et al. 2012), as the ability to detect overarching patterns is complicated by uncertainties about the life history traits of commonly-hunted species (Bowler et al. 2014), variation in their dispersal rates, and the availability of nearby un-hunted areas that can replenish prey stocks in areas that are exploited (Shepard et al. 2012). Nevertheless, there are cases where the presence of hunting has been shown to be a more powerful predictor of mammal abundance than forest type and other extractive activities such as logging (de Thoisy et al. 2005; Marshall et al. 2006; Rovero et al. 2012), and population declines and extirpations of taxa that are popular prey have been commonly recorded in forests surrounding settlements (Peres 1990, 2000; Cullen et al. 2000; Nuñez-Iturri and Howe 2007; Zapata-Ríos et al. 2009; Rosin and Swamy 2013). Forests that have been subject to heavy hunting tend to have significantly less vertebrate biomass and fewer individuals of large-bodied species, particularly tapirs (*Tapirus terrestris* and *Tapirus bairdii)*, white-lipped peccaries (*Tayassu pecari)*, ateline monkeys, tinamous, curassows and trumpeters. Evidence is beginning to show the cascading effects of this absence on tree communities (Nuñez-Iturri and Howe 2007; Terborgh et al. 2008; Stevenson 2011) and invertebrate assemblages (Andresen and Laurance 2007), which may eventually alter the dynamics of whole forests. As human populations continue to increase, there is concern that forest encroachment, better access to markets and the availability of firearms could exacerbate existing problems and eventually lead to the defaunation of forests across large areas. For these reasons, it is important to understand both the structure of hunting profiles across different communities as well as what drives the differences between them.

Similarities among the type of species that are found at lower densities or are absent from areas where heavy hunting takes place suggests that hunters across the neotropics target species in a predictable manner (Peres and Palacios 2007). Ultimately the hunting profile (i.e. which species are hunted in what numbers) of any particular settlement will be the result of a number of influences, dictated by the efficiency of collecting a particular resource, the influences of culture and personal preference, and the likelihood of success of each pursuit. Hunters are principally assumed to act in line with optimal foraging theory; that is, in a way that will give them the maximum return for a given amount of hunting effort (Cowlishaw and Dunbar 2000). This means they are expected to target species that are either large and thus offer the highest return for a successful kill, or that are abundant and easy to harvest. Pacas and agoutis, for example, are tolerant to disturbance and can often be hunted in crop fields and gardens (Redford and Robinson 1987). They can thus be easier to hunt and have relatively short pursuit times compared to larger prey such as monkeys and brocket deer (Alvard 1993). Overlaid upon these parameters, however, will be cultural influences that can alter the value of species targeted. Woolly monkeys (*Lagothrix*), for example, offer good returns in terms of meat because of their large size, but have added prestige as centerpieces for festivals and weddings (Sirén 2012; Stafford et al. 2016). Cultural taboos in neotropical indigenous groups, which may protect some species from hunting, are widespread but inconsistent. For example, in a preliminary review of neotropical indigenous taboos on primates Cormier (2006) found variation in the species that are avoided but also who the taboo applied to, with some restrictions dependent upon age, gender and reproductive status. Taboos can also change over time (e.g. Yost and Kelley 1983; Maldonado Rodríguez 2010) and can be affected by prey scarcity (e.g. Maldonado Rodríguez 2010). Prohibitions on the consumption of species including white-tailed and red brocket deer, tapirs and peccaries, for instance, used to be more widespread in Amazonian indigenous groups but have rapidly broken down (Redford and Robinson 1987).

A third consideration is the ability of a species to persist under varying degrees of hunting pressure, which is usually a function of reproductive rates, location (i.e. whether there any areas that can potentially act as sources of immigration into hunted areas) and abundance (Bodmer et al. 1997). Unfortunately, the large body mass that tends to characterize preferred species often coincides with life history traits that make them highly vulnerable to overexploitation; including low reproductive rates, long lifespans, and long generation times (Bodmer et al. 1997). Ateline primate species, for example, give birth to single young, have long inter-birth periods, and have group structures where not all females may be reproductively active (Cowlishaw and Dunbar 2000). Group size may also be important; for example, Peres (1990) noticed that the large group size and low group density of woolly monkeys in the Brazilian Amazon meant that a single encounter with hunters could be enough to eliminate a large percentage of the total population. Likewise, group ambushes of white-lipped peccary herds have been known to harvest as many as 82 animals in a single hunt (Peres 1996). These features are likely to make some species more vulnerable to overexploitation. As they decline, the composition of hunting profiles is expected to shift away from a small subset species and towards a more diverse profile with a lower average biomass.

The last large cross-site analysis of neotropical hunting profiles, carried out in by Jerozolimski and Peres (2003), investigated whether several generally accepted patterns of hunter behavior could be detected in the harvest lists of 31 tribal and non-tribal sites, from source data published from 1972 to 2000. The authors explored (1) whether larger prey species were preferentially hunted and (2) whether the population size or age of settlements could predict the diversity of species targeted or the average biomass of prey. A regression analysis on the body mass of prey items and the Ivlev’s electivity index for the species hunted at five of the settlements provided strong evidence for large species being preferentially targeted. While they found no significant correlation between a settlement’s size and either the species richness or average biomass of mammals consumed, a settlement’s age was correlated with both the number of species a community targeted (*r* ^2^ = 0.550) and the average biomass of animals that were hunted (*r* ^2^ = 0.171). While the relationship between the mean body mass of mammal kills and the age of settlement was linear (with older settlements hunting lighter species), the relationship between the number of species hunted and a community’s age was quadratic; remaining relatively stable for the first few years of hunting, and only starting to diversify after a settlement had been inhabited for approximately 15 years.

Increasing awareness of prey depletion and sustainability, the introduction of domestic animals such as cattle and poultry that provide readily available protein, and changes in the costs and benefits of different hunting techniques raise an interesting question of whether the patterns of prey preference and depletion detected in Jerozolimski and Peres (2003) are still present in more recent data. Shifts away from more traditional prey species (such as monkeys) and towards species that are more favoured by colonists and were previously subject to taboo (such as red brocket deer) have been recorded in studies that are over 30 years old [Saffirio and Hames (1983) in Redford and Robinson (1987) and Yost and Kelley (1983)], and it is reasonable to expect that these patterns may have become more pronounced in recent years. In this paper, we expand upon Jerozolimski and Peres’ (2003) previous analyses by adding 24 papers containing the hunting profiles of 49 communities published from 1997 to 2012. We compare hunting patterns across Central America, Amazonia and the Guianan shield, aiming to (1) analyze the variability of hunting profiles across different locations, (2) investigate whether communities that are geographically closer have similar hunting profiles, and (3) investigate whether the age and population size of settlements are good predictors of the number of species hunted, the evenness of hunting profiles, and the average biomass of prey.

## Methods

### Data compilation and study criteria

We performed a literature search for hunting profiles of communities throughout the neotropics using the following search criteria: (1) sites must be located in Central America, Amazonia or the Guianan shield, (2) their surrounding areas must be principally covered by moist tropical forest and (3) the study must include the type and number/biomass of mammals hunted over a defined time period. We compiled data from 78 communities (or, in 12 cases, from several communities whose data was pooled together) from 44 papers in 10 countries (Supplementary Table 1). For each study, we collated (1) the study duration, in days (2) the site’s geographic location, using co-ordinates provided in the paper or satellite images to pinpoint the study location on a map, (3) the population size of the settlement when the study was conducted, (4) the age of the settlement when the study was conducted, (5) the number of carcasses recorded by species and (6) the biomass of species extracted, and whether this was actively measured during the study or estimated using literature values. We included 28 of the 31 communities included in Jerozolimski and Peres (2003) but were not able to obtain the offtake data for the remaining three.

**Table 1:** Number of communities for which information was available to carry out regression analyses

Analysis	Number of settlements
Size versus number of mammal species hunted	61
Size versus diversity of the prey profile	60
Size versus average body mass of individuals hunted	54
Age versus number of mammal species hunted (truncated at 25)	44
Age versus number of mammal species hunted (not truncated)	39
Age versus diversity of the prey profile (truncated at 25)	44
Age versus diversity of the prey profile (not truncated)	39
Age versus average body mass of individuals hunted (truncated at 25)	36
Age versus average body mass of individuals hunted (not truncated)	34

### Assumptions

We assume that all our hunting data accurately reflects the true relative contributions of each species to the total harvest, even though different studies use different methods of data collection (hunting diaries and interviews) and not all studies were able to record the activity of all hunters. Studies have noted that hunters are sometimes prone to underreporting small-mammal catches (Smith 1976; Koster 2007; Santos-Fita et al. 2012). Similarly, not every study ran for a full year, and may, therefore, have under-represented species which are only hunted in certain seasons. For example, Franzen’s (2006) study did not cover months that were best for hunting woolly monkeys, but did cover the time of year where white-lipped peccaries congregated around fruiting palms and could be hunted in larger numbers. We also assume that the hunting range of settlements contained species that filled the same range of functional niches, and in the absence of certain genera had an analogous congener. For example, the red brocket deer's (*Mazama americana*) range does not extend to central America, but it is replaced by white-tailed deer (*Odocoileus virginianus*).

### Geographical variation

We investigated geographic variation in hunting profiles firstly by creating a map showing the proportion of individuals in each community’s hunting profile that belonged to each mammalian order. We then generated two distance matrices that measured (a) geographical proximity and (b) hunting profile similarity between all communities. For geographical proximity, we used the geosphere package (Hijmans 2016) in R and the latitude and longitude coordinates of each community to generate a distance matrix using the Vincenty Ellipsoid method, which assumes the shape of the earth is an oblate spheroid. For hunting proximity we used each community’s breakdown of percentage kills according to order to generate a matrix of their hunting profile similarity using a Bray-Curtis dissimilarity index. This was carried out using the vegan package (Oksanen et al. 2016) in R. We then used a Mantel test with 999 permutations to investigate if the two matrices were correlated. Communities which had unknown or only approximate locations were excluded from the analysis, leaving n = 68 settlements. In order to visualize our data, we used the Ward method of hierarchical clustering to generate and visualize trees of our two matrices.

We note that studies which ran for a short time or were conducted in small communities had the potential to introduce heteroscedasticity into our dataset if they were disproportionately influenced by the actions a small proportion of hunters or the events taking place over a short number of study days. We therefore carried out two additional robustness analyses to test whether the inclusion of study effort (defined by the number of days each study ran for) or the community’s population size influenced the outcome of the relationship between a community’s location and its hunting profile. We carried out two partial Mantel tests, including either the length of study in days or the size of a community as a factor. Due to missing data regarding the study length and population size of settlements, n = 65 when study length was included as a factor, and n = 54 when size was included instead.

### Species preferences

We examined species preferences in more detail for the three mammalian orders that were most commonly hunted: primates, rodents, and artiodactyls. All genera were checked for synonyms and standardized using names quoted on the IUCN red list. We excluded from our analysis any communities whose offtake tallies used common names that included an unknown number of genera, e.g. ‘peccary’, ‘monkey’ or ‘deer’. For each community, a genus that was not included in their hunting profile was assigned a value of NA; this is because in the majority of instances we were unable to distinguish between a species that was not listed because it was avoided totally, or because it was not present in the hunting catchment to begin with. For each order, we used a generalized linear mixed effects model with a negative binomial distribution to test for differences in the number of kills among genera, including community as a random effect, in the glmmADMB package in R (Fournier et al. 2012). We tested the model for overdispersion following the approach suggested at http://glmm.wikidot.com/faq, and conducted posthoc pairwise comparisons between genera using the glht function in the multcomp package (Hothorn et al. 2008). In order to avoid having expected values of under 5, we pooled together the small bodied species of primates (*Callithrix, Cebuella, Callicebus, Saimiri* and *Saguinus*) in our primate dataset, and *Proechimys* and *Orthogeomys* (spiny rats and pocket gophers) in our rodent dataset. We also excluded *Blastocerus* (marsh deer) from our artiodactyl dataset, as only a single individual was hunted over our whole dataset.

### Correlates of offtake profiles

We initially checked whether the number of species hunted was associated with study length, as longer studies might be expected to produce more diverse profiles because they are more likely to include species that are hunted only rarely. This was tested using Spearman’s rank correlation analysis with n = 72 communities. We excluded four communities whose offtake tallies used common names that included an unknown number of genera.

We used the same proxies for hunting pressure used in Jerozolimski and Peres (2003) (i.e. the age of communities and their population size) to test for relationships in three different aspects of hunting profiles: (a) the number of species targeted, (b) the Simpson’s diversity index of the offtake profile, and (c) the average body mass of species hunted, calculated by summing the total number of individuals recorded in the hunting profile and dividing by their total, undressed biomass. We consider a diversity index to be a potentially more informative metric than the number of species hunted as it includes a measurement of the evenness of the profile. Hunting profiles of younger settlements may be expected to have low values of diversity (and high value of the index, *D*) if a few preferred species make up the bulk of animals hunted. However, as these become depleted, a more even profile would be expected and the value of *D* would be expected to decrease.

During data collection it became clear that there was a large amount of missing data regarding the age and/or size of communities, as well as the data each study recorded regarding its hunting profile. Not all communities, for example, recorded the biomass of kills, whereas others recorded common instead of scientific names of prey items and some used blanket terms such as ‘large monkey’ which could potentially include multiple genera. In light of this we did not analyze the effect of a community’s age and size on our three chosen hunting metrics together in a general linear model, instead performing separate regression analyses for the age and size of settlements versus our three chosen hunting profile metrics. Table 1 shows the number of studies that were available for each analysis. In particular, there was a lot of missing data regarding the exact age of settlements. Jerozolimski and Peres (2003) addressed this problem by truncating a community’s maximum age at 25 years, so in order to perform a comparison with our expanded dataset we also truncated our settlement ages at 25 before carrying out a regression analysis. However, our new dataset included settlements that were up to 70 years old; we therefore also ran the analysis on an un-truncated dataset to check that the patterns detected were not an artifact of truncation.

## Results

### Overview

We recorded a total of 90 named species across 78 communities, belonging to 11 mammalian orders (Carnivora (15 spp.), Cetartiodactyla (9 spp.), Chiroptera (unknown number of species), Cingulata (5 spp.), Didelphimorphia (1 sp.), Lagomorpha (2 sp.), Perissodactyla (2 spp.), Pilosa (7 spp.), Primates (35 spp.), Rodentia (13 spp.) and Sirenia (1 sp.)). The average number of mammal species hunted was 13, although hunting profiles ranged from just five species recorded at an unnamed Sanemá Community in Bolívar, Venezuela, to 26 species recorded at Sarayaku in eastern Ecuador. Cetartiodactyla and Rodentia were the most widely hunted orders, with carcasses from both orders recorded in 100% of offtake lists. Primates, carnivores, perrisodactyls and cingulatans appeared in 83, 65, 65 and 61% of lists, respectively. Members of the order Pilosa were hunted more rarely, occurring in just under one-third of lists, whereas lagomorphs (represented by two species, *Sylvilagus braisliensis* and *Sylvilagus floridanus*) only appeared in 14% and didelphimorphia (also a single species, *Didelphis marsupialis*) in 3%. Sirenians only occurred in one hunting profile (a Siona settlement in Cuyabeno National Park, Ecuador) whereas Chiroptera were only recorded in the hunting profile of a Hupdu Maku settlement in Brazil (the species was not specified).

### Geographic patterns

**Fig. 1:**
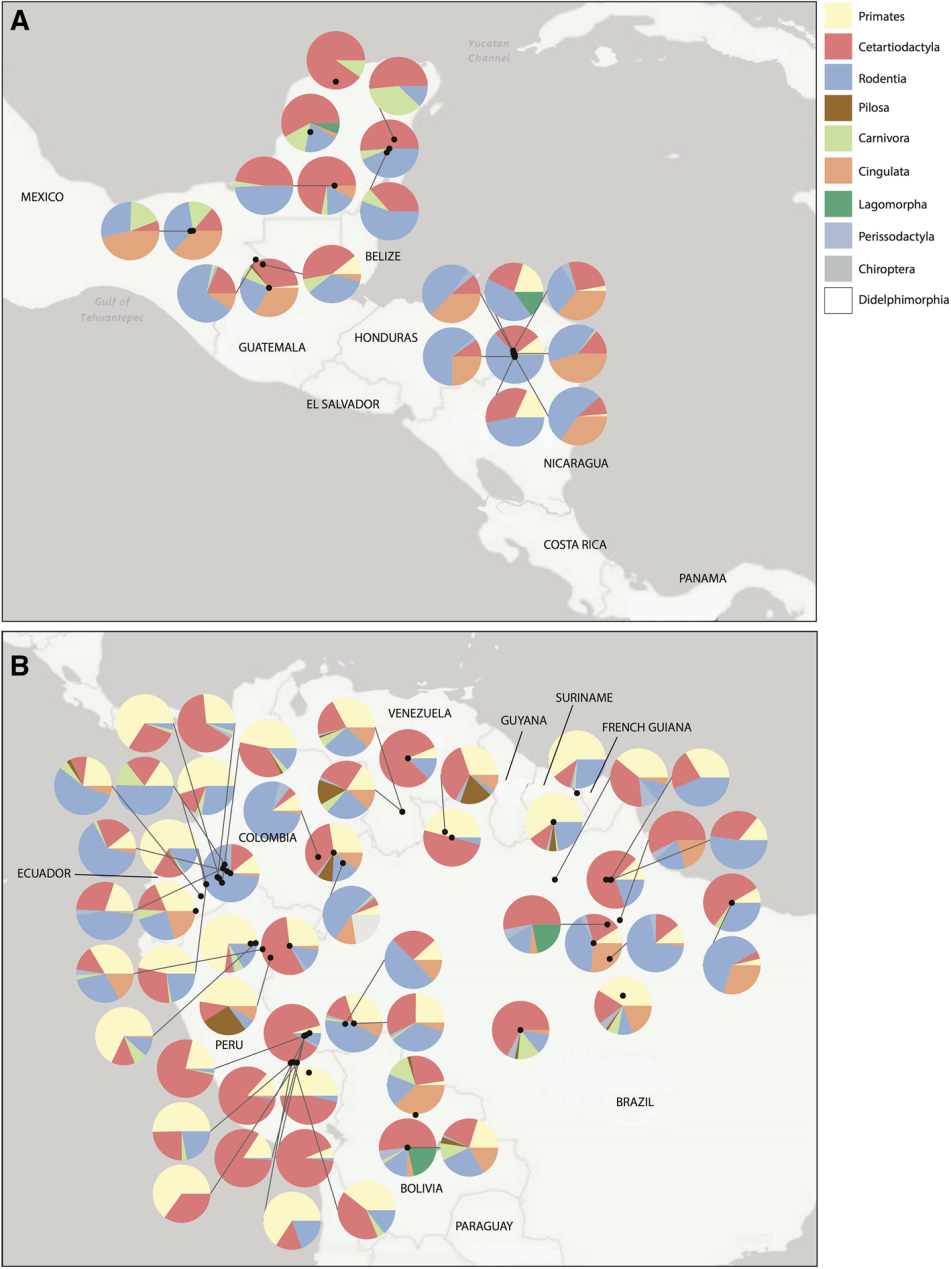
Proportion of kills in n = 75 settlements by mammal order in **a** Central America and **b** South America. *Black dots* show the real location of each settlement

Figure 1 shows the proportion of kills belonging to each mammalian order for the communities in our dataset. Cetartiodactyls, rodents and primates are the three orders that dominate profiles, though the latter were not as prevalent in hunting profiles in central America and were almost completely absent from the profiles of communities in Mexico. Cingulata were generally more prevalent in Central America than in South America, though there are a number of exceptions. Pilosa were only hunted in substantial numbers (i.e. they accounted for over 5% of catches) in four communities (Toropo-teri, Venezuela; Unnamed Matses Community, Peru; Group of Bara Maku Settlements, Colombia & Uxiutheri/Iropitheri/Maxipiutheri, Brazil), which is not surprising given that the meat of sloths and anteaters is widely considered to taste bad (Koster 2008; Parathian and Maldonado 2010; Quiroga et al. 2016). Carnivores (including coatis (*Nasua*), by far the most commonly hunted genus of that order) were hunted throughout the geographic range of our sample.

**Fig. 2:**
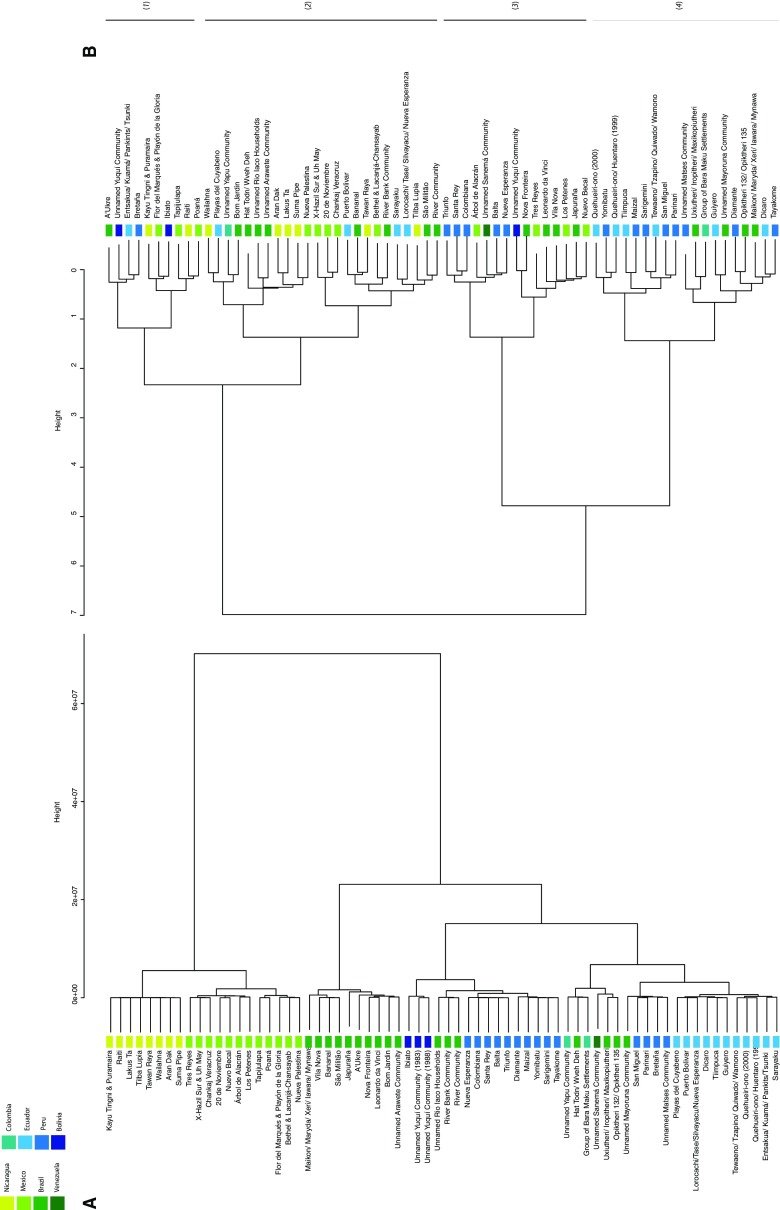
A comparison of trees showing the relationships of settlements according to **a** geographical proximity and **b** similarity of hunting profile according to the percentage of mammals hunted belonging to each order. Trees were generated using hierarchical clustering (Ward method)

Figure 2 shows a comparison of trees that were generated according to (a) the geographical proximity of settlements to one another and (b) the similarity of their hunting profiles according to the percentage of individuals hunted belonging to different mammalian orders. There was a significant but weak positive correlation between the location of communities and their hunting profiles (Mantel test, *r* = 0.1597, *P* = 0.001, *n* = 68). Including the survey effort and the size of communities in partial Mantel tests on the smaller datasets for which all information was available made little difference to the relationship (for survey effort *n* = 65, *r* = 0.1774, *P* = 0.01, for size *n* = 54, *r* = 0.2372, *P* = 0.001). The geographic tree shows a split between central American settlements located in Mexico and Nicaragua and those located in the Amazon and the Guianan shield. Within the second split there are a cluster of settlements in central Brazil, another with settlements from Venezuela, Colombia and northern Brazil, another from northern Peru and Ecuador, and a final group with settlements from southern Peru, south western Brazil and Bolivia. These groupings are not conserved in the offtake similarity tree, though Mexican and Nicaraguan settlements tend to be placed on one side of a main split and Peruvian and Ecuadorian settlements tend to be placed on the other (other countries were split fairly evenly between the two). Nevertheless there are a large number of exceptions to this pattern, and settlements that clustered closely together geographically could have dramatically different hunting profiles. For example, the profile of Wailahna, a Mayanga community situated in Jinotega, Nicaragua, was most similar to Playas del Cuyabeno, Kichwa community in Sucumbios, Ecuador. Within the hunting tree’s first split there are two main clusters of settlements: one group is characterized by high numbers of armadillos and rodents as well as low numbers of primates (see (1) on Fig. 2); whereas the other is characterized by high numbers of rodent kills (2). In the second main split, settlements cluster together that have a focus on either cetartiodactyls (3) or primates (4).

### Species preferences

**Fig. 3:**
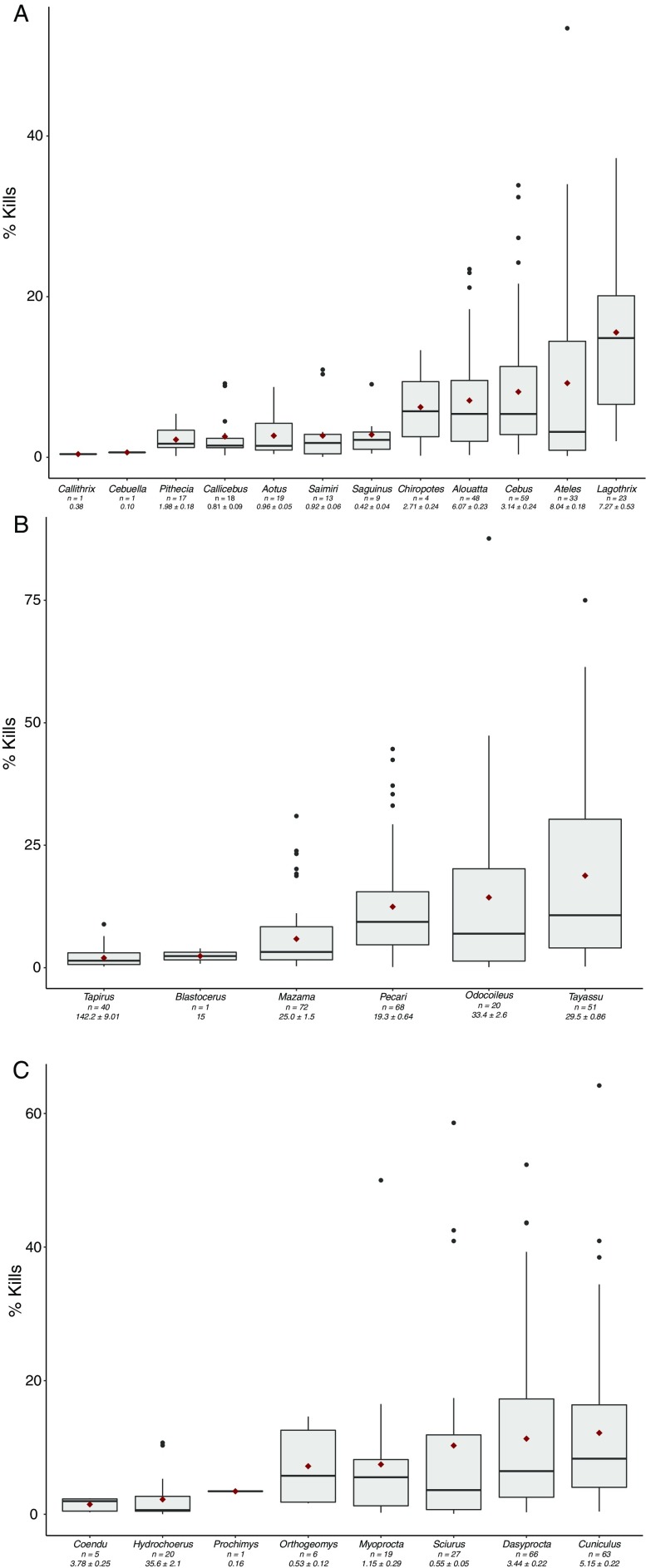
Genus preferences for the three most commonly hunted mammalian orders, **a** Primates, **b** Artiodactyls, and **c** Rodents. For each genus, the plots show data only from profiles where at least one animal belonging to it was included in the list of kills. The number of prey profiles featuring each genus is given below the genus name, alongside the average biomass of each genus (±SE), calculated from all studies which weighed carcasses and did not use literature values. *Red dots* show the average percentage kills for each genus

Figure 3 shows the percentage of total kills recorded for each genus in the three most popular orders targeted by hunters (primates, artiodactyls and rodents), alongside their average weight and the number of hunting profiles where the genus was featured. The boxplots use data only from lists where a minimum of one individual of that genus was hunted (i.e. they do not include zeroes). This was because for the majority of genera we were unable to distinguish between zeroes recorded as a result of total avoidance, or zeroes that were due to the absence of a genus from a hunting catchment. In primates, the two largest genera accounted on average for the largest percentage of kills in our hunting profiles, although *Lagothrix* (woolly monkeys) outstrips *Ateles* (spider monkeys), the latter of which accounted for the widest range (0.7–53%) of percentage kills. *Cebus* (capuchins) on average accounted for a higher percentage of kills than *Alouatta* (howler monkeys), despite their smaller size. The two other medium-bodied genera, *Pithecia* and *Chiropotes* (sakis and bearded-sakis) accounted for lower percentages of total kills, with the average for the former being much closer to the smaller *Callicebus* (titis), *Saguinus* (tamarins) and *Saimiri* (squirrel monkeys). The mean percentage of total kills for *Chiropotes* was higher but much more variable. Table 2 shows the pairwise comparisons of differences between genera for each order using the raw count data of hunting profiles. Significant differences were recorded between *Lagothrix* and smaller and medium bodied genera (except *Chiropotes*). Numbers of *Pithecia* hunted were also significantly different from the four most popular genera in terms of the average percentage of kills they accounted for (*Lagothrix, Ateles, Alouatta* and *Cebus).*

**Table 2:** Posthoc pairwise comparisons of the number of kills for each genus belonging to the primates, artiodactyls and rodents

Order	Comparison	Estimate	SE	Z value	Pr (>|z|)
Primates	*Lagothrix*–*Ateles*	0.740	0.384	1.926	0.486
	*Lagothrix*–*Cebus*	0.918	0.351	2.617	0.129
	*Lagothix*–*Alouatta*	0.920	0.266	3.454	0.011*
	*Lagothrix*–*Chiropotes*	1.738	0.640	2.718	0.100
	*Lagothrix*–*Aotus*	1.847	0.407	4.535	<0.001***
	*Lagothrix*–*Pithecia*	−2.056	0.399	−5.147	<0.001***
	*Lagothrix*–*Small Primates*	−1.187	0.400	−2.963	0.052
	*Ateles*–*Cebus*	−0.178	0.340	−0.523	0.999
	*Ateles*–*Alouatta*	0.180	0.270	0.666	0.997
	*Ateles*–*Chiropotes*	−0.998	0.638	−1.565	0.732
	*Ateles*–*Aotus*	1.107	0.433	2.556	0.149
	*Ateles*–*Pithecia*	−1.315	0.417	−3.152	0.030*
	*Ateles*–*Small Primates*	−0.446	0.416	−1.072	0.951
	*Cebus*–*Alouatta*	0.002	0.217	0.010	1.000
	*Cebus*–*Chiropotes*	−0.821	0.595	−1.378	0.839
	*Cebus*–*Aotus*	0.929	0.396	2.347	0.235
	*Cebus*–*Pithecia*	−1.138	0.379	−3.000	0.046*
	*Cebus*–*Small Primates*	−0.269	0.386	−0.697	0.996
	*Alouatta*–*Chiropotes*	−0.818	0.575	−1.423	0.815
	*Alouatta*–*Aotus*	−0.927	0.314	−2.949	0.054
	*Alouatta*–*Pithecia*	−1.136	0.302	−3.759	0.004**
	*Alouatta*–*Small Primates*	−0.267	0.303	−0.880	0.983
	*Chiropotes*–*Aotus*	0.109	0.671	0.162	1.000
	*Chiropotes*–*Pithecia*	−0.317	0.648	−0.490	1.000
	*Chiropotes*–*Small Primates*	0.552	0.656	0.841	0.987
	*Aotus*–*Pithecia*	−0.209	0.423	−0.493	1.000
	*Aotus*–*Small Primates*	0.660	0.409	1.614	0.701
	*Pithecia*–*Small Primates*	0.869	0.417	2.086	0.380
Artiodactyls	*Tayassu*–*Odocoileus*	0.184	0.408	0.449	0.990
	*Tayassu*–*Pecari*	0.320	0.296	1.080	0.793
	*Tayassu*–*Mazama*	0.941	0.219	4.289	<0.001***
	*Tayassu*–*Tapirus*	2.027	0.324	6.259	<0.001***
	*Odocoileus*–*Pecari*	−0.136	0.374	−0.365	0.995
	*Odocoileus*–*Mazama*	0.757	0.338	2.239	0.147
	*Odocoileus*–*Tapirus*	−1.843	0.425	−4.341	<0.001***
	*Pecari*–*Mazama*	0.621	0.193	3.219	0.010**
	*Pecari*–*Tapirus*	−1.707	0.319	−5.351	<0.001***
	*Mazama*–*Tapirus*	−1.086	0.244	−4.461	<0.001***
Rodents	*Cuniculus*–*Dasyprocta*	−0.243	1.009	−0.241	1.000
	*Cuniculus*–*Sciurus*	−0.085	1.068	−0.080	1.000
	*Cuniculus*–*Myoprocta*	−0.635	1.064	−0.596	0.996
	*Cuniculus*–*Orthogeomys/Prochimys*	−1.116	1.092	−1.021	0.939
	*Cuniculus*–*Hydrochoerus*	−1.825	1.079	−1.692	0.588
	*Cuniculus*–*Coendu*	1.669	0.732	2.280	0.231
	*Dasyprocta*–*Sciurus*	0.158	1.087	0.146	1.000
	*Dasyprocta*–*Myoprocta*	−0.391	1.024	−0.382	1.000
	*Dasyprocta*–*Orthogeomys/Prochimys*	−0.872	1.160	−0.752	0.986
	*Dasyprocta*–*Hydrochoerus*	−1.581	1.083	−1.460	0.740
	*Dasyprocta*–*Coendu*	1.426	0.727	1.962	0.407
	*Sciurus*–*Myoprocta*	0.549	1.087	0.505	0.998
	*Sciurus*–*Orthogeomys/Prochimys*	1.030	1.195	0.862	0.973
	*Sciurus*–*Hydrochoerus*	1.740	1.103	1.577	0.665
	*Sciurus*–*Coendu*	1.584	0.769	2.061	0.347
	*Myoprocta*–*Orthogeomys/Prochimys*	−0.481	1.224	−0.393	1.000
	*Myoprocta*–*Hydrochoerus*	1.190	1.067	1.115	0.909
	*Myoprocta*–*Coendu*	1.035	0.773	1.339	0.809
	*Orthogeomys/Prochimys*–*Hydrochoerus*	0.709	1.207	0.588	0.996
	*Orthogeomys/Prochimys*–*Coendu*	0.554	0.904	0.613	0.995
	*Hydrochorus*–*Coendu*	−0.155	0.777	−0.200	1.000

Differences were calculated using three generalized linear mixed effect models with a negative binomial distribution, including community as a random effect. Posthoc comparisons were generated using the glht function in the R package multcomp (Hothorn et al. 2008)

* *P* < 0.05, ** *P* < 0.01, *** *P* < 0.001

Cetartiodactyls accounted for an average of 11% of kills in n = 78 offtake profiles. *Tayassu* (white-lipped peccaries), *Pecari* (collared peccaries) and *Odocoileus* (white-tailed deer) were the three genera that accounted for, on average, the highest percentage of total mammalian kills. Comparisons using raw count data (including *Tapirus*, a perissodactyl) showed that numbers of *Tayassu*, the most popular genus in terms of the average percentage of kills it accounted for, were significantly different from *Mazama* and *Tapirus*, but not *Odocoileus* and *Pecari*. Rodents accounted for an average of 9% of kills. Our analysis of pairwise differences from our generalized mixed linear model showed no differences between genera in terms of raw counts of animals hunted for this order. In terms of the percentage of average kills, the most popular genera were *Cuniculus* (pacas) followed by *Dasyprocta* (agoutis)*, Sciurus* (squirrels), *Myoprocta* (acouchis) and *Orthogeomys* (pocket gophers). Despite being the heaviest rodent, *Hydrochoerus* (capybara) accounted for, on average, a very low percentage of kills.

### Correlates of offtake profiles

#### Length of study

**Fig. 4:**
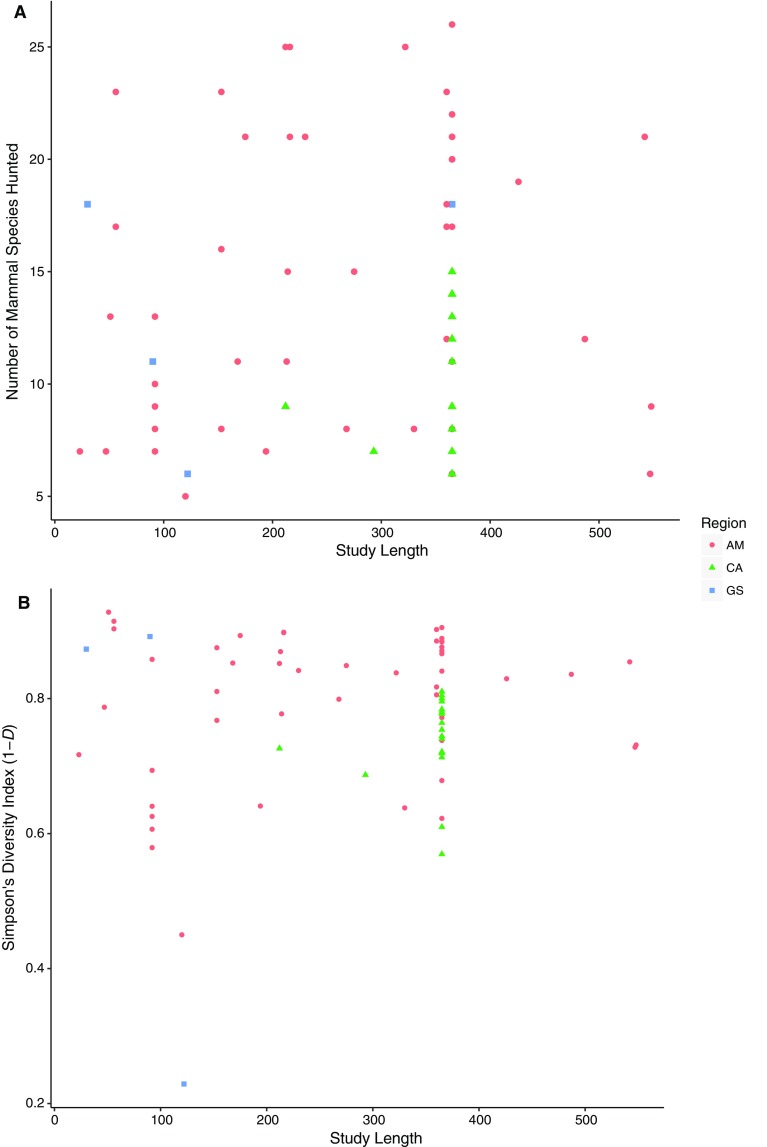
Study length, in days, versus **a** the number of species recorded in hunting profiles and **b** the Simpson’s diversity index score of offtake profiles (1−*D*). *CA* Central America, *GS* Guianan Shield, *AM* Amazonia

There was no significant relationship between the length of studies included in our analysis and the number of species recorded (Spearman’s rank correlation *r*_*s*_ = 0.04, *P* = 0.79, *n* = 72) or the Simpson’s diversity index of offtake profiles (Spearman’s rank correlation, *r*_*s*_ = −0.07, *P* = 0.57, *n* = 71) (Fig. 4). This suggests that the studies included in our analyses ran for a sufficient amount of time for the number of species recorded in each community to reach a plateau. Thus the offtake profiles included in our analysis are likely to be representative of the true diversity of species targeted.

#### Population size of settlement

**Fig. 5:**
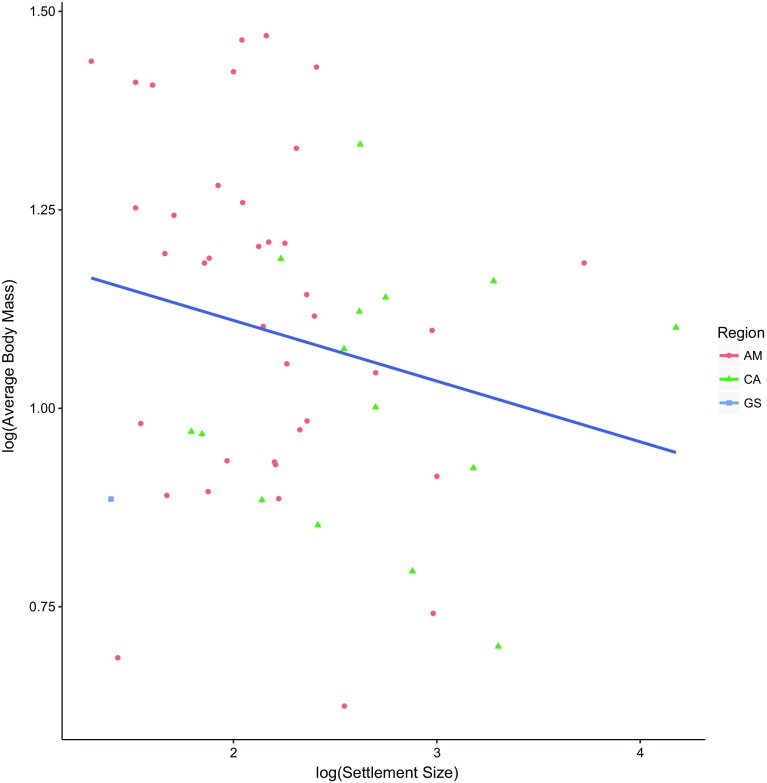
Average body mass of kills vs. settlement size (*n* = 54). *CA* Central America, *GS* Guianan Shield, *AM* Amazonia

The population size of settlements was not related to either the number of species hunted (linear model, *r* ^2^ = 0.01, *n* = 61, *P* = 0.418) or Simpson’s diversity value of hunting profiles (linear model, *r* ^2^ = 0.02, *n* = 60, *P* = 0.263). There was also no correlation between a settlement’s size and the average body size of species hunted (linear model, *r* ^2^ = 0.04, *n* = 54, *P* = 0.121) (Fig. 5).

#### Age of settlement

**Fig. 6:**
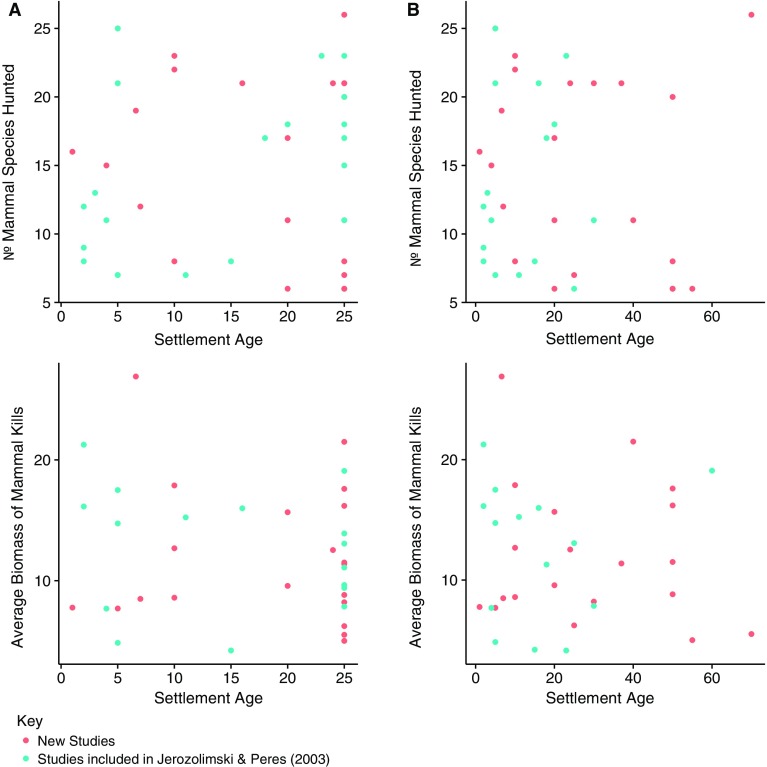
The relationship between the age of settlements and (1) the number of mammals hunted and (2) the average biomass of animals hunted. Column **a** shows results when the maximum age is truncated at 25, column **b** shows results with no truncation when only settlements with a known age are included

Figure 6 shows the number of species hunted versus the age of settlements, where their maximum age is truncated at 25. Unlike Jerozolimski and Peres (2003) we did not find evidence of hunters diversifying their hunting portfolio after 15 years; instead our data indicate that there is no relationship between a settlement’s age and the number of species included in its hunting profile (linear model, *r* ^2^ = 0.002, n = 44, *P* = 0.769). An untruncated dataset including older settlements whose age is known, but discarding those where the exact age was unknown gives similar results. Similarly we did not find any significant relationship between a settlement’s age and its diversity index in either a truncated (*r* ^2^ = 0.000, n = 44, P = 0.892) or an untruncated dataset (linear model, *r* ^2^ = 0.002, n = 39, P = 0.783). We also did not find any correlation between age of settlements and the average biomass of species hunted on a truncated dataset ((linear model, *r* ^2^ = 0.02, *n* = 36, *P* = = 0.416), or an untruncated dataset that allowed for the full range of settlement ages (*r* ^2^ = 0.007, *n* = 34, *P* = 0.645) (Fig. 6).

## Discussion

### Preferences

The predominance of rodents and cetartiodactyls across the geographic range of our sample indicates that these two orders are the cornerstones of prey provision for hunters in neotropical communities. Primates were much more prominent in the profiles of South American communities than in Central American ones. It is possible that this difference was, in part, caused by the absence of *Lagothrix* in Central America, which is the most popular primate genus when it is present in a hunting profile; but this would be difficult to confirm from the available data. Other ateline primates (*Ateles* and *Alouatta*) are present in these areas but were only hunted in large numbers in four communities, indicating that the lack of *Lagothrix* does not mean that other atelines are more heavily targeted to compensate for their absence. Our data also support the commonly accepted pattern of larger and medium-bodied primates being preferentially hunted, with the exception of *Pithecia* that, on average, made up a similar percentage of kills to smaller species such as *Saimiri* and *Aotus*.

We were surprised that *Sciurus* was the third most popular rodent genus in terms of the percentage of hunting kills they account for in communities that hunt them, when they are seldom included in lists of preferred rodent prey species. As the largest rodent species, capybaras also accounted for a surprisingly low average percentage of kills in the 20 settlements where they were recorded; however, some communities where preferences have been studied consider their meat to be lean and have poor taste (Sirén 2004; Koster et al. 2010; Pinheiro and Moreira 2012).

There are of course a number of problems with comparing preferences by combining hunting data from different communities that span a time range of 40 years. Regardless of a settlement’s age, cultural shifts over this timeframe in the Amazonian basin have been enormous (Roosevelt 2013); but these are unlikely to have been consistent over the whole area. Additionally, not every community will be starting out with the same availability of game, and different forests could be able to replace game at different rates depending on both their intrinsic productivity and their proximity to un-hunted areas that could act as a source of new animals (Novaro et al. 2000; Shepard et al. 2012). Each profile could therefore show preferences under different conditions, which may be constrained by circumstance and could theoretically change if certain species were more abundant. Species diversity is not homogenous across all of Latin America (Tognelli and Kelt 2004; Schipper et al. 2008), and communities situated in more diverse areas may be expected to hunt a larger range of species, especially if there are several closely-related variations of an animal that is a preferred prey type (for example, *Dasypus kappleri/Dasypus novemcinctus,* or *Mazama americana/Mazama gouazoubira*). Focusing solely on mammals also ignores the potential influence of the availability of bird and reptile meat, the abundance of which has the potential to increase or decrease the reliance on certain mammal species; in particular, several settlements relied heavily on tortoises [(e.g. Bom Jardin and the unnamed Arawete community in Milton (1991)]. Ideally, long-term longitudinal studies would be able to evaluate changes in the hunting profiles of communities while controlling for forest productivity, but there are very few which have run for a significant time span and these cover an extremely limited geographical range. Our study also highlights the lack of accurate hunting catchment assessments in hunting studies and the lack of estimations of the proportion of the population that actively hunt, which would allow for a truer estimation of hunting pressure.

### Geographical variation and correlates of prey profiles

We had expected the hunting profiles of each community to be strongly correlated with how close they were to one another. This is because the forests hunted by communities that were closer together were assumed to hold similar starting populations of species and communities were more likely to have cultural exchange and thus foster similar preferences and taboos. Our data showed that the geographical proximity of settlements and the similarity of their hunting profiles was significantly correlated, but only weakly. Generally, settlements that were in Central America could be found on one side of principal split in the hunting profile tree. However, communities that were close together could have conspicuously different hunting offtake profiles, which implies either that (a) cultural preferences can vary greatly over short distances or that (b) the availability of prey species is highly variable over short distances. Forest productivity is known to vary across the South America with implications for both species richness (Kay et al. 1997) and abundance (Emmons 1984; Haugaasen and Peres 2005), but it is difficult to know whether these differences operate on a scale that would account for the differences seen in hunting profiles. Because we could not find any relationship between the age or size of settlements and the three metrics of prey profiles we tested them against (the number of mammal species hunted, the Simpson’s diversity index of the prey profile and the average body mass of species hunted) we think it unlikely that the differences seen are a product of some areas starting out with depleted prey availability due to hunting activities.

Some of the results of our analysis contrast with those found in Jerozolimski and Peres (2003). Notably, we did not find any relationship between the average body mass of species hunted or the number of species hunted and a settlement’s age, and did not find that communities started to diversify the number of species targeted after approximately 15 years. Some of the communities included in our analysis were over 20 years old but still exhibited very selective prey harvests, which suggests that they have not needed to diversify as a result of diminishing returns from preferred prey species. These results are encouraging from a conservation perspective; but are at odds with studies that have repeatedly found evidence of prey depletion in heavily hunted areas (Peres 1990, 2000; Cullen et al. 2000; Nuñez-Iturri and Howe 2007; Rosin and Swamy 2013). It is possible that we are simply using the wrong proxies, and that the age and size of settlements do not accurately reflect actual hunting pressure; but studies of hunting activity often do not include estimates of metrics that would allow this to be investigated (the size of the hunting catchment or the proportion of the settlements population that actively hunt, for example).

## Conclusions

Our results generally agree with previous studies showing that larger genera are preferentially hunted, but that for some genera the relationship is not always proportional and/or predictable. Woolly monkeys, for example, generally account for a higher percentage of kills in hunting profiles than would be predicted by their body size. Similarly, *capybaras* only account for a very low average percentage of kills across profiles where they were featured; possibly protected by the bad taste of their meat. Unlike previous studies we find no relationship between a settlement’s size and age and the number of mammal species hunted, the diversity of its hunting profile, or the average size of prey being brought back to the community. We conclude that a community’s age and population size are poor proxies of actual hunting pressure.

Finally, our results show that hunting profiles change substantially over short distances, raising an important question of whether their dissimilarity is driven by differences in forest productivity over very small scales or rapidly diverging cultural preferences. Either way, our results highlight the importance of tailoring conservation programs to the needs of each community, and that ‘one-size fits all’ interventions, even targeted to close neighbors, may not necessarily address the right species or practices.

## Electronic supplementary material

Below is the link to the electronic supplementary material.

Supplementary material 1 (DOCX 30 kb) Supplementary Table 1 List of communities used in our study. Dashes are used where data was not available. *Studies whose precise location is unknown, †Precise location unknown, but approximate co-ordinates assigned from descriptions in the paper for the purposes of showing the data in Fig. 1. ‡Study did not contain the number of individuals hunted, but gave information on the total biomass of each species extracted

Supplementary material 2 (DOCX 79 kb) Supplementary Table 2 List of mammal species recorded in the combined hunting profiles of n = 78 communties
