# Supplementary material for: A pan-neotropical analysis of hunting preferences
Source: Biodivers Conserv. 2017 Apr 25;26(8):1877–97. doi: 10.1007/s10531-017-1334-8 (PMC6979659; doi:10.1007/s10531-017-1334-8)
Supplement: Supplementary file 1 — Supplementary material 1 (DOCX 30 kb) Supplementary Table 1 List of communities used in our study. Dashes are used where data was not available. *Studies whose precise location is unknown, †Precise location unknown, but approximate co-ordinates assigned from descriptions in the paper for the purposes of showing the data in Fig. 1. ‡Study did not contain the number of individuals hunted, but gave information on the total biomass of each species extracted [file 10531_2017_1334_MOESM1_ESM.docx]

| **Settlement Number*(Paper Number)*** | **Community** | **Group** | **Location** | **Settlement Age/ Pop size** | **Study Duration (days)** | **Source** |
| --- | --- | --- | --- | --- | --- | --- |
| 1 *(1)* | Los Petenes | Maya | Campeche, Mexico | -/ - | 212 | León Martinez 2006 |
| 2 *(2)* | 20 de Noviembre | Maya | Campeche, Mexico | - / 350 | 365 | Santos-Fita et al. 2012 |
| 3 *(2)* | Nuevo Becal | Mestizo | Campeche, Mexico | 40 / 420 | 365 | Santos-Fita et al. 2012 |
| 4 *(3)* | Bethel &Lacanjá-Chansayab | Lacandon | Chiapas/Yucatán, Mexico | -/ 560 | 365 | Naranjo et al. 2004 |
| 5 *(3)* | Nueva Palestina | Tzeltal | Chiapas/Yucatán, Mexico | -/ 15000 | 365 | Naranjo et al. 2004 |
| 6 *(3)* | Flor del Marqués&Playón de la Gloria | Mestizo | Chiapas/Yucatán, Mexico | -/ 500 | 365 | Naranjo et al. 2004 |
| 7 *(4)* | X-Hazil Sur & Uh May | Maya | Quintana Roo, Mexico | -/ 1902 | 365 | Santos-Fita et al. 2012 |
| 8 (*4*) | Chankaj Veracruz | Maya | Quintana Roo, Mexico | -/ 416 | 365 | Santos-Fita et al. 2012 |
| 9 *(5)* | Tres Reyes | Maya | Quintana Roo, Mexico | -/ 150 | 293 | Quijano-Hernández &Calmé 2002 |
| 10 *(6)* | Tapijulapa | Unknown | Tabasco, Mexico | 55/ 2005 | 365 | Contreras-Moreno et al. 2012 |
| 11 *(6)* | Poaná | Unknown | Tabasco, Mexico | 25/ 759 | 365 | Contreras-Moreno et al. 2012 |
| 12 *(7)* | Árbol de Alacrán | Maya | Yucatán, Mexico | 20/ 3027 | 122 | MontielOrtega et al. 2000 |
| 13 *(8)* | AranDak | Mayanga | Jinotega, Nicaragua | -/ 260 | 365 | Williams-Guillén et al. 2006 |
| 14 *(8)* | KayuTingni&Puramaira | Miskito | Jinotega, Nicaragua | -/ 171 | 365 | Williams-Guillén et al. 2006 |
| 15 *(8)* | Lakus Ta | Miskito | Jinotega, Nicaragua | -/ - | 365 | Williams-Guillén et al. 2006 |
| 16 *(8)* | Raiti | Miskito | Jinotega, Nicaragua | -/ 1513 | 365 | Williams-Guillén et al. 2006 |
| 17 *(8)* | Suma Pipe | Miskito | Jinotega, Nicaragua | -/ 70 | 365 | Williams-Guillén et al. 2006 |
| 18 *(8)* | Tawan Raya | Mayangna/Miskito | Jinotega, Nicaragua | -/ 62 | 365 | Williams-Guillén et al. 2006 |
| 19 *(8)* | TilbaLupia | Mayangna/Miskito | Jinotega, Nicaragua | -/ - | 365 | Williams-Guillén et al. 2006 |
| 20 *(8)* | Wailahna | Mayanga | Jinotega, Nicaragua | -/ 138 | 365 | Williams-Guillén et al. 2006 |
| 21 *(9)* | Toki† | Ye'kwana | Amazonas, Venezuela | 5/ 76 | 216 | Hames& Vickers 1982, Hames 1979 |
| 22 *(9)* | Toropo-teri† | Yanomamo | Amazonas, Venezuela | 5/ 51 | 51 | Hames& Vickers 1982, Hames 1979 |
| 23*(10)* | Unnamed Sanemá Community | Sanemá | Bolívar, Venezuela | -/ 33 | 120 | Sponsel 1981 |
| 24*(11)* | Unnamed Tirio Community† | Tirio | Sipaliwini, Suriname | 20/ 450 | 30 | Lenselink 1972 |
| 25*(12)* | Unnamed Suralco Community† | Suralco | Suriname (region unspecified) | 4/ 25 | 89 | Mittermeier 1991 |
| 26*(13)* | Zidock‡ | Wayapi | French Guyana | -/ 212 | 365 | Ouhoud-Renoux 1998 |
| 27*(14)* | A'Ukre | Kayapo | Pará, Brazil | 16/ 133 | 542 | Peres &Nascimento 2006 |
| 28 *(15)* | Maikon/Maryda/Xeri/Iawara/Mynawa | WaimiriAtroari | Pará, Brazil | 7/ 256 | 426 | de Souza Mazurek et al. 2000 |
| 29*(16)* | Nova Fronteira | Mestizo | Pará, Brazil | 2/ 204 | 360 | Smith 1976 |
| 30*(16)* | Leonardo da Vinci | Mestizo | Pará, Brazil | 2/ 179 | 268 | Smith 1976 |
| 31*(16)* | Coco Chato* | Mestizo | Pará, Brazil | 15/ 361 | 330 | Smith 1976 |
| 32*(17)* | Unnamed Arawete Community | Arawete | Pará, Brazil | -/ - | - | Milton 1991 |
| 33*(17)* | BomJardin | Parakana | Pará, Brazil | -/ - | - | Milton 1991 |
| 34*(17)* | Unnamed Mayoruna Community | Mayoruna | Pará, Brazil | -/ - | - | Milton 1991 |
| 35*(18)* | Unnamed Rio Iaco Households | Mestizo | Acre, Brazil | - (Over 25)/ 405 | 360 | Martins 1993 |
| 36*(19)* | River Bank Community | Mestizo | Acre, Brazil | - (Over 25)/ 229 | 360 | Calouro 1995 |
| 37*(19)* | River Community | Mestizo | Acre, Brazil | - (Over 25)/ 212 | 360 | Calouro 1995 |
| 38*(20)* | Bananal | Non-tribal | Amapá, Brazil | 50/ - | 365 | Parry et al. 2009 |
| 39*(20)* | São Militão | Non-tribal | Amapá, Brazil | 50/ - | 365 | Parry et al. 2009 |
| 40*(20)* | Vila Nova | Non-tribal | Amapá, Brazil | 50/ - | 365 | Parry et al. 2009 |
| 41*(21)* | Japuranã | Colonist | MatoGrosso, Brazil | 4/ - | 214 | Trinca& Ferrari 2004 |
| 42*(22)* | Site 1† | Ka'apor | Maranhão, Brazil | 5/ 27 | 23 | Balée 1985 |
| 43*(22)* | Site 2† | Ka'apor | Maranhão, Brazil | 11/ 72 | 47 | Balée 1985 |
| 44*(23)* | Uxiutheri/Iropitheri/Maxikopiutheri (traditional villages) | Yanomami | Roraima, Brazil | 3/ 67 | 51 | Saffirio&Scaglion 1982 |
| 45*(23)* | Opiktheri 132/Opiktheri 135 (acculturated villages) | Yanomami | Roraima, Brazil | 5/ 74 | 194 | Saffirio&Scaglion 1982 |
| 46*(24)* | Hat Todn/ WvehDeh* | HupduMaku | Brazil | 30/ 75 | 168 | Reid 1979 |
| 47*(25)* | Group of Bara Maku Settlements* | Bara Maku | Colombia | 60/ 84 | 275 | Silverwood-Cope 1972 |
| 48*(26)* | Yapu | Tatuyo- Yapú | Vaupés, Colombia | 14/ 110 | 107 | Dufour 1981 |
| 49*(27)* | Joint 4 | Shuar | Morona Santiago, Ecuador | 30/ 1000 | 365 | Zapata-Rios et al. 2009 |
| 50*(28)* | Tewaeno/ Tzapino/ Quiwado/ Wamono | Waorani | Orellana, Ecuador | -/ 230 | 275 | Yost & Kelley 1983 |
| 51*(29)* | Quehueiri-ono/ Huentaro | Waorani | Napo, Ecuador | 7/ 161 | 487 | Lu 1999 |
| 52*(30)* | Quehueiri-ono | Waorani | Napo, Ecuador | 5/ 167 | 322 | Mena et al. 2000 |
| 53*(31)* | Tiimpuca | Waorani | Orellana, Ecuador | 1/ 47 | 153 | Franzen et al. 2006 |
| 54*(31)* | Guiyero | Waorani | Orellana, Ecuador | 10/ 33 | 153 | Franzen et al. 2006 |
| 55*(31)* | Dicaro | Waorani | Orellana, Ecuador | 10/ 140 | 153 | Franzen et al. 2006 |
| 56*(32)* | Sarayaku | Kichwa | Pastaza, Ecuador | 70/ 960 | 365 | Siren 2004 |
| 57*(33)* | Playas del Cuyabeno | Kichwa | Sucumbios, Ecuador | -/ 111 | 213 | PrietoAlbuja2011 |
| 58*(33)* | Puerto Bolivar | Siona | Sucumbios, Ecuador | -/ 159 | 212 | PrietoAlbuja2011 |
| 59*(34)* | San Pablo de Shushufundi† | SionaSecoya | Sucumbios, Ecuador | 2/ 132 | 548 | Vickers 1980 |
| 60*(35)* | Lorocachi/Tasé/Silvayacu/Nueva Esperanza | Kichwa | Sucumbios, Ecuador | 24/ 948 | 175 | Zapata Ríos 2001 |
| 61*(36)* | Sabalo* | Cofán | Sucumbios, Ecuador | 10/ 93 | - | Schel 1997 |
| 62*(37)* | San Miguel | Unknown | Loreto, Peru | -/ - | 365 | Aquino &Calle 2003 |
| 63*(37)* | Parinari | Unknown | Loreto, Peru | -/ - | 365 | Aquino &Calle 2003 |
| 64*(38)* | Bretaña | Unknown | Loreto, Peru | -/ 5298 | 92 | Saldaña&Saldaña 2011 |
| 65*(39)* | Unnamed Matses Community | Matses | Loreto, Peru | 7/ 549 | 240 | Romanoff 1984 |
| 66*(40)* | Tayakome | Matsigenka | Madre de Dios, Peru | 42/ 149 | 365 | Ohl- Schacherer et al. 2007 |
| 67*(40)* | Yomibatu | Matsigenka | Madre de Dios, Peru | 37/ 183 | 365 | Ohl- Schacherer et al. 2007 |
| 68*(40)* | Sarigemini | Matsigenka | Madre de Dios, Peru | 14/ 35 | 365 | Ohl- Schacherer et al. 2007 |
| 69*(40)* | Maizal | Matsigenka | Madre de Dios, Peru | 14/ 46 | 365 | Ohl- Schacherer et al. 2007 |
| 70*(41)* | Diamante | Piro | Madre de Dios, Peru | - (Over 25)/ 250 | 547 | Alvard 1993 |
| 71*(42)* | Santa Rey | Cashinahua | Ucayali, Peru | -/ 100 | 92 | Gil Navarro et al. 2004 |
| 72*(42)* | Balta | Cashinahua | Ucayali, Peru | -/ 110 | 92 | Gil Navarro et al. 2004 |
| 73*(42)* | Triunfo | Cashinahua | Ucayali, Peru | -/ 40 | 92 | Gil Navarro et al. 2004 |
| 74*(42)* | Colombiana | Cashinahua | Ucayali, Peru | -/ 20 | 92 | Gil Navarro et al. 2004 |
| 75*(42)* | Nueva Esperanza | Cashinahua | Ucayali, Peru | -/ 145 | 92 | Gil Navarro et al. 2004 |
| 76*(43)* | Unnamed Yuquí Community | Yuqui (1983) | Cochabamba, Bolivia | -/ - | 56 | Stearman 1990 |
| 77*(43)* | Unnamed Yuquí Community | Yuqui (1988) | Cochabamba, Bolivia | -/ - | 56 | Stearman 1990 |
| 78 (*44)* | Ibiato | Sirionó | Beni, Bolivia | (Over 25)/ 500 | 360 | Townsend 2000 |

Supplementary Table 1. List of communities used in our study. Dashes are used where data was not available. *Studies whose precise location is unknown, †Location unknown, but co-ordinates assigned from descriptions in the paper for the purposes of showing the data in Figure 1. ‡Study did not contain the number of individuals hunted, but gave information on the total biomass of each species extracted.

References:

Alvard MS (1993) Testing the “ecologically noble savage” hypothesis: Interspecific prey choice by Piro hunters of Amazonian Peru. Biotropica 39:41–146

Aquino R, Calle A (2003) Evaluación del estado de conservación de los mamíferos de caza : un modelo comparativo en comunidades de la Reserva Nacional Pacaya Samiria (Loreto, Peru). Rev Peru Biol 10:163–174.

Balée W (1985) Ka’apor Ritual Hunting. Hum Ecol 13:485–510.

Calouro AM (1995) Caça de subsistência: sustentabilidade e padrões de uso entre seringueiros ribeirinhos e não-ribeirinhos do Estado do Acre. Dissertation, University of Brasilia

Contreras-Moreno FM, De la Cruz-Félix K, Bello-Gutiérrez J (2012) Uso patrones de cacería y preferencia de presas en dos sitios del Parque Estatal La Sierra, Tabasco, México. Etnobiología 10:1–9.

De Souza-Mazurek RR, Pedrinho T, Feliciano X, et al (2000) Subsistence hunting among the Waimiri Atroari Indians in central Amazonia, Brazil. Biodivers Conserv 9:579–596. doi: 10.1023/A:1008999201747

Dufour DL (1981) Household variation in energy flow in a population of tropical forest horticuluralists. Doctoral Thesis, State University of New York at Binghamton

Franzen M (2006) Evaluating the sustainability of hunting: a comparison of harvest profiles across three Huaorani communities. Environ Conserv 33:36–45. doi: 10.1017/S0376892906002712

Gil Navarro T, Francisco Puricho M, López del Aguila C, Roque Moreyra P, Herrera Sarmiento J (2004) Aprovechamiento de la Fauna Silvestre en Comunidades Cashinahua del Río Curanja y Purús. WWF. http://www.portalces.org/biblioteca/areas-protegidas/aprovechamiento-de-fauna-silvestre-comunidades-cashinahua-del-rio. Accessed 9 April 2016

Hames RB (1979) Comparison of the efficiency of the shotgun and the bow and arrow in neotropical forest hunting. Hum Ecol 7:219–252.

Hames RB, Vickers WT (1982) Optimal diet breadth theory as a model to explain variability in Amazonian hunting. Am Ethnol 9:358–378. doi: 10.1525/ae.1982.9.2.02a00090

Lenselink J (1972) De Jachtopbrengst in een Surinaams Trio-dorp. De Surinaamse Landbouw, Paramaribo 20:37-41

León Martínez PN (2006) Aprovechamiento de fauna silvestre en una comunidad aledaña a la Reserva de la Biosfera Los Petenes, Campeche. Dissertation, Centro de Investigación y de Estudios Avanzados del Instituto Politécnico Nacional

Lu FE (1999) Changes in Subsistence Patterns and Resource Use of the Huaorani Indians in the Ecuadorian Amazon. Doctoral Thesis, University of North Carolina

Martins E (1993) A caça de subsistência de extrativistas na Amazônia: sustentabilidade, biodiversidade e extinção de espécies. Dissertation, University of Brasilia

Mena VP, Stallings JR, Regalado BJ, Cueva LR (2000) The sustainability of current hunting practices by the Huaorani. In: Robinson JG, Bennett EL (eds) Hunting for Sustainability in Tropical Forests. Columbia University Press, New York, pp 57-78

Milton K (1991) Comparative aspects of diet in Amazonian forest-dwellers. Philos Trans R Soc Lond B Biol Sci 334:253–263, discussion 263. doi: 10.1098/rstb.1991.0114

Mittermeier RA (1991) Hunting and is effects on wild primate populations in Suriname. In Robinson JG, Redford KH (eds) Neotropical Wildlife Use and Conservation. University of Chicago Press, London, pp 93-107

Montiel Ortega SM, Arias Reyes LM, Dickinson F (2000) La caceria tradicional en el norte de Yucatán: una práctica comunitaria. Rev Geogr Agrícola 43–52.

Naranjo EJ, Guerra MM, Bodmer RE, Bolanos J (2004) Subsistence hunting by three ethnic groups of the Lacandon forest, Mexico. J Ethnobiol 24:233–253.

Ohl-Schacherer J, Shepard GH, Kaplan H, et al (2007) The sustainability of subsistence hunting by Matsigenka native communities in Manu National Park, Peru. Conserv Biol 21:1174–1185. doi: 10.1111/j.1523-1739.2007.00759.x

Ouhoud-Renoux F (1998) Se nourrir a Trois-Sauts: analyse diachronique de la predation chez les Wayapi de Haut Oyapock (Guyane francaise). Journal d'agriculture traditionelle et de botanique appliqué 1-2:181-206

Parry L, Barlow J, Peres CA (2009) Hunting for sustainability in tropical secondary forests. Conserv Biol 23:1270–80. doi: 10.1111/j.1523-1739.2009.01224.x

Peres CA., Nascimento HS (2006) Impact of game hunting by the Kayapó of south-eastern Amazonia: implications for wildlife conservation in tropical forest indigenous reserves. Biodivers Conserv 15:2627–2653. doi: 10.1007/s10531-005-5406-9

Prieto F (2011) Densidad de Mamiferos y Aves de Caza en el Territorio Siona dePuerto Bolivar y su situacion actual en el Rio Cuyabeno, Reserva de Produccion Faunistica Cuyabeno, Amazonia Ecuatoriana. Dissertation, Pontifica Universidad Católica del Ecuador (PUCE)

Quijano-Hernández E, Calmé S (2002) Patrones De Caceria Y Conservación De La Fauna Silvestre En Una Comunidad Maya De Quintana Roo. Etnobiología 2:1–18.

Reid H (1979) Some Aspects of Movement Growth and Change among the Hupdu Maku Indians of Brazil. Doctoral Thesis, University of Cambridge

Romanoff SA (1984) Matses Adaptations in the Peruvian Amazon. Doctoral Thesis, Columbia University

Saffirio G, Scaglion R (1982) Journal of Anthropological Research,. J Anthropol Res 38:315–327.

Saldaña, S & Saldaña V (2011) La cacería de animales silvestres en la comunidad de Bretaña, Río Puinahua, Loreto – Perú. Revista Colombiana de Ciencia Animal 3:225-237

Santos-Fita D, Naranjo EJ, Rangel-Salazar JL (2012) Wildlife uses and hunting patterns in rural communities of the Yucatan Peninsula, Mexico. J Ethnobiol Ethnomed 8:38. doi: 10.1186/1746-4269-8-38

Schel L (1997) Cacería de vertebrados terrestres y recomendaciones para organizar los esfuerzos de conservación de especies en la comunidad Cofán de Zábalo, Reserva de Producción Faunística Cuyabeno, Amazonía Ecuatoriana. Dissertation, Pontificia Universidad Católica del Ecuador (PUCE)

Silverwood- Cope P (1972) A Contribution to the Ethnography of the Colombian Maku. Doctoral Thesis, University of Cambridge

Sirén A (2004) Changing Interactions between Humans and Nature in Sarayaku , Ecuadorian Amazon. Doctoral Thesis, Swedish University of Agricultural Sciences Uppsala

Smith NJH (1976) Utilization of game along Brazil’s transamazon highway. Acta Amazonica 6:455-466

Sponsel LE (1981) The Hunter and the Hunted in the Amazon: An Integrated Biological and Cultural Approach to the Behavioral Ecology of Human Predation. Doctoral Thesis, Cornell University

Stearman AM (1990) The Effects of Settler Incursion on Fish and Game Resources of the Yuquí, a Native Amazonian Society of Eastern Bolivia. Hum Organ 4:373-385

Townsend WR (2000) The sustainability of subsistence hunting by he Sirionó Indians of Bolívia. In: Robinson JG, Bennett EL (eds) Hunting for Sustainability in Tropical Forests. Columbia University Press, New York, pp 267-281

Trinca CT, Ferrari SF (2003) Caça em assentamento rural na Amazônia mato-grossense. http://www.anppas.org.br/encontro_anual/encontro2/GT/GT02/GTCristiano.pdf Accessed 04 April 2016

Vickers WT (1980) An analysis of Amazonian hunting yields as a function of settlement age. Working Papers on South American Indians 2:7-29

Williams-Guillén K, Griffith D, Polisar J, et al (2006) Abundancia de animales de caza y características de cacería en el territorio indígena de Kipla Sait Tasbaika, Reserva de Biosfera BOSAWAS. Wani 46:37–61.

Yost JA, Kelley PM (1983) Shotguns, blowguns and spears: the analysis of technological efficiency. In: Hames RB, Vickers ET (eds) Adaptive Responses of Native Amazonians. Academic Press, New York, pp189-224

Zapata-Ríos G, Urgilés C, Suárez E (2009) Mammal hunting by the Shuar of the Ecuadorian Amazon: is it sustainable? Oryx 43:375–385. doi: 10.1017/S0030605309001914

Zapata Ríos G (2001) Sustentabilidad de la cacería de subsistencia: el caso de cuatro comunidades quichuas en la Amazonía nororiental ecuatoriana. Mastozoología Neotrop / J Neotrop Mammal 8:59–66.
