# Supplementary material for: A pan-neotropical analysis of hunting preferences
Source: Biodivers Conserv. 2017 Apr 25;26(8):1877–97. doi: 10.1007/s10531-017-1334-8 (PMC6979659; doi:10.1007/s10531-017-1334-8)
Supplement: Supplementary file 2 — Supplementary material 2 (DOCX 79 kb) Supplementary Table 2 List of mammal species recorded in the combined hunting profiles of n = 78 communties [file 10531_2017_1334_MOESM2_ESM.docx]

| **Scientific Name** | |  | **Common name** |
| --- | --- | --- | --- |
| **Carnivora** |  | |  |
| *Bassaricyon* | *alleni* | | Eastern lowland olingo |
| *Eira* | *barbara* | | Tayra |
| *Herpailurus* | *yagouaroundi* | | Jaguarundi |
| *Leopardus* | *pardalis* | | Ocelot |
|  | *wiedii* | | Margay |
| *Nasua* | *narica* | | White-nosed coati |
|  | *nasua* | | South American coati |
| *Nasuella* | *olivacea* | | Western mountain coati |
| *Panthera* | *onca* | | Jaguar |
| *Potos* | *flavus* | | Kinkajou |
| *Procyon* | *cancrivorus* | | Crab-eating raccoon |
|  | *lotor* | | Northern raccoon |
| *Pteronura* | *brasiliensis* | | Giant otter |
| *Puma* | *concolor* | | Puma |
| *Tremarctos* | *ornatus* | | Spectacled bear |
| **Cetartiodactyla** |  | |  |
| *Blastocerus* | *dichotomus* | | Marsh deer |
| *Mazama* | *rufina* | | Dwarf red brocket deer |
|  | *pandora* | | Yucatan brown brocket deer |
|  | *americana* | | Red brocket deer |
|  | *gouazoubira* | | Gray brocket deer |
|  | *nemorivaga* | | Amazonian brown brocket deer |
| *Odocoileus* | *virginianus* | | White-tailed deer |
| *Pecari* | *tajacu* | | Collared peccary |
| *Tayassu* | *pecari* | | White-lipped peccary |
| **Chiroptera** |  | |  |
| Species not specified | | |  |
| **Cingulata** |  | |  |
| *Cabassous* | species not specified | | Armadillo |
| *Dasypus* | *kappleri* | | Greater long-nosed armadillo |
|  | *novemcinctus* | | Nine-banded armadillo |
| *Euphractus* | *sexcinctus* | | Six-banded armadillo |
| *Priodontes* | *maximus* | | Giant armadillo |
| **Didelphimorphia** | | |  |
| *Didelphis* | *marsupialis* | | Common opossum |
| **Lagomorpha** |  | |  |
| *Sylvilagus* | *brasiliensis* | | Tapeti |
|  | *floridanus* | | Eastern cottontail |
| **Perissodactyla** |  | |  |
| *Tapirus* | *terrestris* | | Lowland tapir |
|  | *bairdii* | | Baird's tapir |
| **Pilosa** |  | |  |
| *Bradypus* | *tridactylus* | | Pale-throated three-toad sloth |
|  | *variegatus* | | Brown-throated sloth |
| *Cyclopes* | *didactylus* | | Silky anteater |
| *Choloepus* | *didactylus* | | Linné's two-toed sloth |
| *Myrmecophaga* | *tridactyla* | | Giant anteater |
| *Tamandua* | *mexicana* | | Northern tamandua |
|  | *tetradactyla* | | Southern tamandua |
| **Primates** |  | |  |
| *Alouatta* | *belzebul* | | Red-handed howler monkey |
|  | *macconnelli* | | Guinana red howler monkey |
|  | *palliata* | | Mantled howler monkey |
|  | *pigra* | | Yucatán black howler monkey |
|  | *seniculus* | | Venezuelan red howler monkey |
| *Ateles* | *belzebuth* | | White-bellied spider monkey |
|  | *paniscus* | | Guiana spider monkey |
|  | *geoffroyi* | | Geoffroy's spider monkey |
|  | *chamek* | | Black-faced black spider monkey |
| *Aotus* | *azarae* | | Azara's night monkey |
|  | *nigriceps* | | Balck-headed night monkey |
|  | *vociferans* | | Noisy night monkey |
| *Cacajao* | *species not specified* | | Uacari |
| *Callicebus* | *cupreus* | | Coppery titi monkey |
|  | *moloch* | | Red-bellied titi monkey |
|  | *torquatus* | | Collared titi monkey |
| *Cebuella* | *pygmaea* | | Pyhmy marmoset |
| *Cebus* | *albifrons* | | White-fronted capuchin |
|  | *apella*  *capucinus* | | Brown capuchin  White-faced capuchin |
|  | *olivaceus* | | Wedge-capped capuchin |
| *Chiropotes* | *satanas* | | Balck bearded saki |
| *Lagothrix* | *lagothrica* | | Common woolly monkey |
|  | *poeppigii* | | Poepigg's woolly monkey |
| *Mico* | *leucippe* | | Golden-white bare-ear marmoset |
| *Pithecia* | *irrorata* | | Rio Tapajós saki |
|  | *monachus* | | Monk saki |
|  | *pithecia* | | White-faced saki |
| *Saimiri* | *bolivensis* | | Black-capped squirrel monkey |
|  | *sciureus* | | Common squirrel monkey |
| *Saguinus* | *fuscicollis* | | Saddleback tamarin |
|  | *imperator* | | Emperor tamarin |
|  | *midas* | | Golden-handed tamarin |
|  | *nigricollis* | | Black mantle tamarin |
|  | *tripartitus* | | Golden-mantle saddleback tamarin |
|  |  | |  |
| ***Rodentia*** |  | |  |
| *Coendou* | *bicolor* | | Bicolor-spined porcupine |
|  | *prehensilis* | | Brazilian porcupine |
| *Cuniculus* | *paca* | | Paca |
| *Dasyprocta* | *fuliginosa* | | Black agouti |
|  | *punctata* | | Central american agouti |
|  | *variegata* | | Brown agouti |
| *Myoprocta* | *acouchy* | | Red acouchi |
|  | *pratti* | | Green acouchi |
| *Hydrochoerus* | *hydrochaeris* | | Capybara |
| *Orthogeomys* | *hispidus* | | Hispid pocket gopher |
| *Sciurus* | *igniventris* | | Northern Amazon red squirrel |
|  | *spadiceus* | | Southern Amazon red squirrel |
|  | *yucatanensis* | | Yucatan squirrel |
| **Sirenia** |  | |  |
| *Trichechus* | *inunguis* | | South American manatee |

**Supplementary Table 2** List of mammal species recorded in the combined hunting profiles of n = 78 communties.
